# Mechanically tunable resins based on acrylate-based resin for digital light processing (DLP) 3D printing

**DOI:** 10.1038/s41598-022-24667-8

**Published:** 2022-11-21

**Authors:** Aphiwat Pongwisuthiruchte, Stephan T. Dubas, Chuanchom Aumnate, Pranut Potiyaraj

**Affiliations:** 1 Department of Materials Science, Faculty of Science, Chulalongkorn University, Bangkok, Thailand; 2 Petroleum and Petrochemical College, Chulalongkorn University, Bangkok, Thailand; 3 Metallurgy and Materials Science Research Institute, Chulalongkorn University, Bangkok, Thailand; 4 Center of Excellence in Responsive Wearable Materials, Chulalongkorn University, Bangkok, Thailand

**Keywords:** Engineering, Materials science

## Abstract

Until now, only a few materials are available for additive manufacturing technologies that employ photopolymerization, such as stereolithography (SLA) and digital light processing (DLP) 3D printing systems. This study investigates a newly formulated resins as an alternative 3D printing materials with tunable mechanical properties to expand the potential applications of advanced engineering products such as wearable devices and small reactors. A commercial acrylate-based resin was selected as a standard resin (STD). The resin was formulated by combining various volume ratios of a low-cost polypropylene glycol (PPG) having various molecular weights (400, 1000, and 2000 g/mol) with the STD resin. The printability of the formulated resins was optimized using the digital light processing (DLP) 3D printing technique. The effects of the PPG contents on the properties of the printed parts were studied, including printability, thermal properties, mechanical properties, and thermo-mechanical properties. As a result, the formulated resins with 5–30%vol of PPG could be printed while higher PPG content led to print failure. Results suggest that increasing the PPG contents reduced the dimensional accuracy of the printed parts and decreased the mechanical properties, including the flexural strength, flexural modulus, impact strength, hardness, and elastic modulus. interestingly, at small loading, 5%vol, the mechanical performance of the printed specimens was successfully enhanced. These results are intriguing to use a tunable mechanical acrylate-based resin for a specific application such as a microreactor.

## Introduction

Wearable and flexible devices such as electronic sensors and transparent flexible displays have attracted a wide range of scientific research and industry interests. Most flexible substrates used today are composed of some polymers such as poly(ethylene terephthalate) (PET), thermoplastic polyurethane (TPU), and poly(dimethylsiloxane) (PDMS), which are typically prepared by the manual assembly of cast segments. However, complex sensing systems are challenging to fabricate with this traditional method, restricting their applications. Three-dimensional (3D) printing technologies have been introduced as an alternative intelligence manufacturing technology to create complex products with ease, low cost, high flexibility, and fast production speed, surpassing traditional manufacturing techniques. Due to the sizeable expanding popularity of the technology, 3D printing, or additive manufacturing (AM) has become increasingly popular for manufacturing both pre-production prototypes and end-user products^1–4^. In several application domains, such as wearable electronics, energy storage, and biomedical devices, 3D printing technologies have been employed extensively. An essential effort required to create 3D printing as a viable production technology is to expand the accessible polymers, particularly high-performance materials, focusing on developing printable materials with tunable properties, for example, conductivity or elasticity^5–10^. As a result, materials for 3D printing are still being studied and developed, particularly those with adjustable features like mechanical performance, conductivity and elasticity.

Vat photopolymerization additive systems (also known as stereolithography (SLA) and digital light projection (DLP)) are among the earliest commercially accessible forms of additive manufacturing technologies. The light source in SLA printing is a single ultraviolet laser beam that draws a model's shape onto the photopolymer resin vat using computer-aided design (CAD) software^11^ and changes the location or angle of optical elements like lenses or mirrors. UV light is used to photochemically solidify, forming a single layer of the desired model^12–15^. The printable materials or resins need to be sufficiently transparent to insure light penetration, while simultaneously avoiding excessive light diffusion that would lead to a loss of resolution^16^. Commercially available resins are primarily formulated for general 3D printing applications such as prototyping rather than explicitly focused on the need for flexible wearable devices. Those existing materials are not necessarily formulated to meet the requirements of flexible devices, particularly the limitation of the flexibility and potential mechanical range for the 3D printed products^17^. However, to develop new printable materials, it is crucial to keep the viscosity of the printable resin low to avoid print failures during DLP 3D printing. Lower viscosity is necessary for rapid printing to allow a new layer of liquid resin to flow into the small gap between the bottom surface of the resin tray and the previous cured solid layer and to maintain dispersion of resin additives^18^.

Polypropylene glycol (PPG) is a low to medium range molar mass polyether with hydroxyl group as the end-group^19^. Due to its viscous colorless and odorless liquid has been utilized in thermoset composites, food chemistry, food processing equipment, cosmetics, pharmaceuticals, and biodegradable polyurethane^20^. It is often used as a plasticizer to reduce the glass transition temperature and improve ductility and processability of brittle biopolymers such as chitosan and polylactic acid (PLA)^21, 22^. Also, PPG is typically used as a precursor to synthesize polyurethane acrylate-based polymers^23–25^.

In this study, the custom formulation of a low-cost UV-curable acrylate-based resin that allows simple mechanical property adjustment with little or no changes to the additive system is presented. Instead of using the acrylate variants (i.e., polypropylene glycol diacrylate), that may increase the crosslinking point and make the resin more rigid^26^ PPG, a low-cost polyester, was selected to improve flexibility and reduce the cost of the commercial UV-curable acrylate-based resin. The printing quality and performance of the formulated resin on DLP 3D printing were compared with those of a commercial counterpart. Furthermore, the formulated resin characteristics, including UV absorbability, chemical functionality, thermal stability, morphology, and mechanical properties, were investigated.

## Results

### Resin formulation

**Figure 1:**
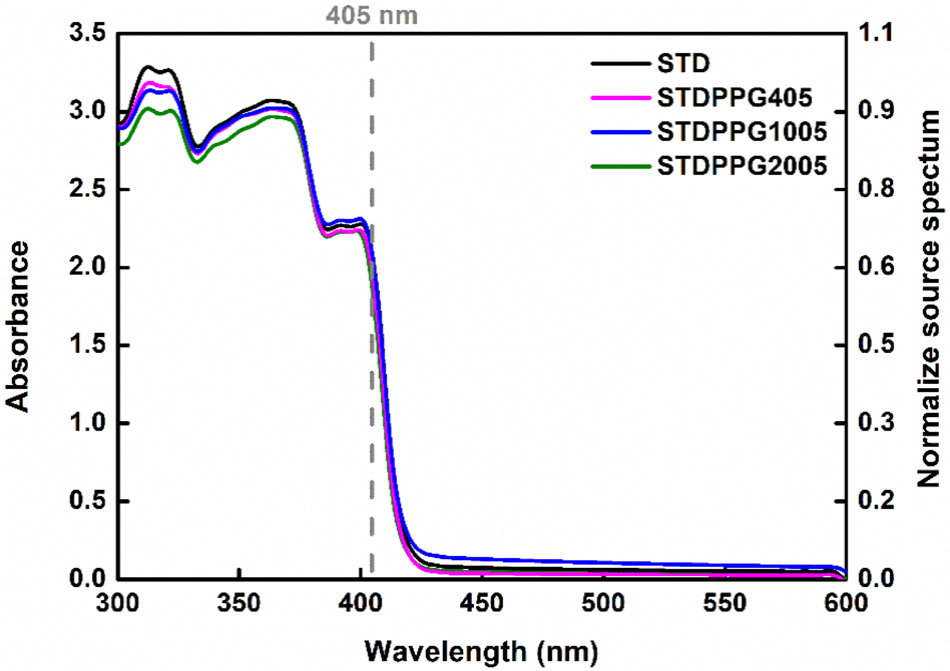
UV absorbance of UV-curable acrylate-based resin (STD) and the formulated resin with PPG400, PPG1000 and PPG2000 at 5%vol added.

Homogeneous UV-curable acrylate-based resins with various PPG content were prepared. For the application to the DLP 3D printing process, the light-absorbing ability of the resins is a crucial parameter. The UV–VIS absorbance spectra of UV-curable acrylate-based resin (STD) and the formulated resins with PPG (5%vol) are shown in Fig. 1. The absorbing ability of all resins was responsive to the UV exposure range of the DLP 3D printer, which was typically in a range of 385–405 nm. Moreover, the results showed an almost identical UV absorbance of the STD and all formulated resins, indicating that the presence of PPG does not affect the UV absorbing ability of the UV-curable acrylate-based resin (STD). Thus, all formulated resins were expected to be 3D printable using the conventional DLP 3D printers.

### Thermogravimetric analysis (TGA)

**Figure 2:**
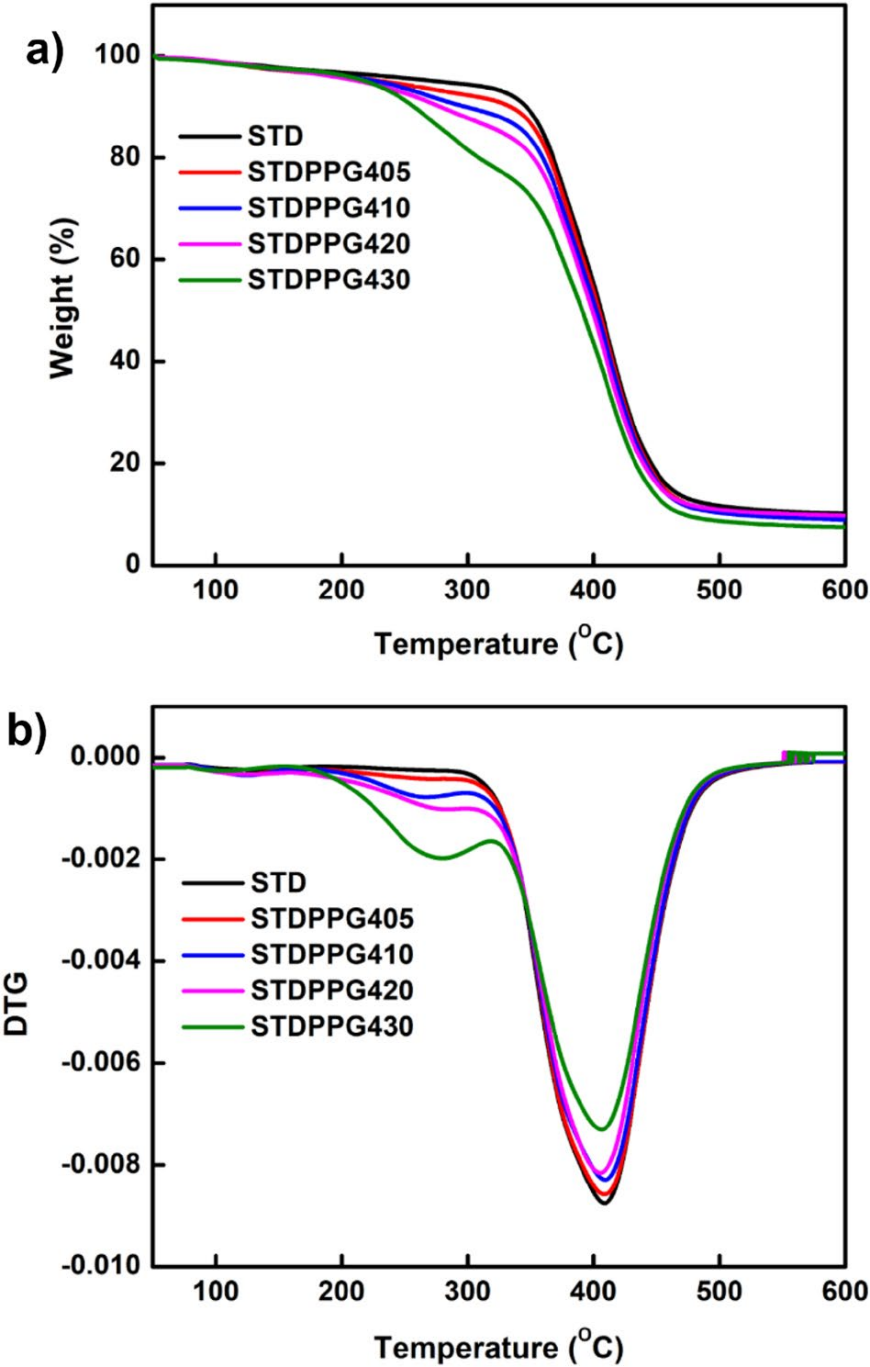
(**a**) Thermogravimetric analysis (TGA) curves and (**b**) derivative thermogravimetric analysis (DTG) curves of UV-curable acrylate-based resin (STD) and formulated resins with PPG400.

To identify the effect of PPG on the thermal stability of the UV-curable acrylate-based resin, thermal gravimetric analysis (TGA) was conducted. Figure 2. shows the TGA thermogram of UV-curable acrylate-based resin (STD) and formulated resins with PPG400 at 5–30%vol added. All formulated resins began to decompose at around 330 °C and ended around 470 °C, with a residual mass of around 9% at 600° C. The decomposition of crosslinking bonds and the residue of carbonaceous formed is attributed to the weight loss around 330–470°C^27–29^. For the formulated resins with PPG1000 and PPG 2000, increasing PPG loading had no significant effect on the thermal stability of the formulated resin, as evidenced by an almost identical TGA thermogram of the UV-curable acrylate-based resin and the formulated resins (see Figs. S2 and S3). However, it should be noted that the effect of PPG loading was significant for the formulated resin with short chain length PPG400. As shown in Fig. 2, the thermal stability of the formulated resin reduced as the PPG loading increase, which could be attributed to the low molecular weight of PPG400 due to the degradation of the PPG structure between 190 and 330 °C^30^.

### Stereolithographic 3D printing of formulated UV-curable acrylate/PPG resins

**Figure 3:**
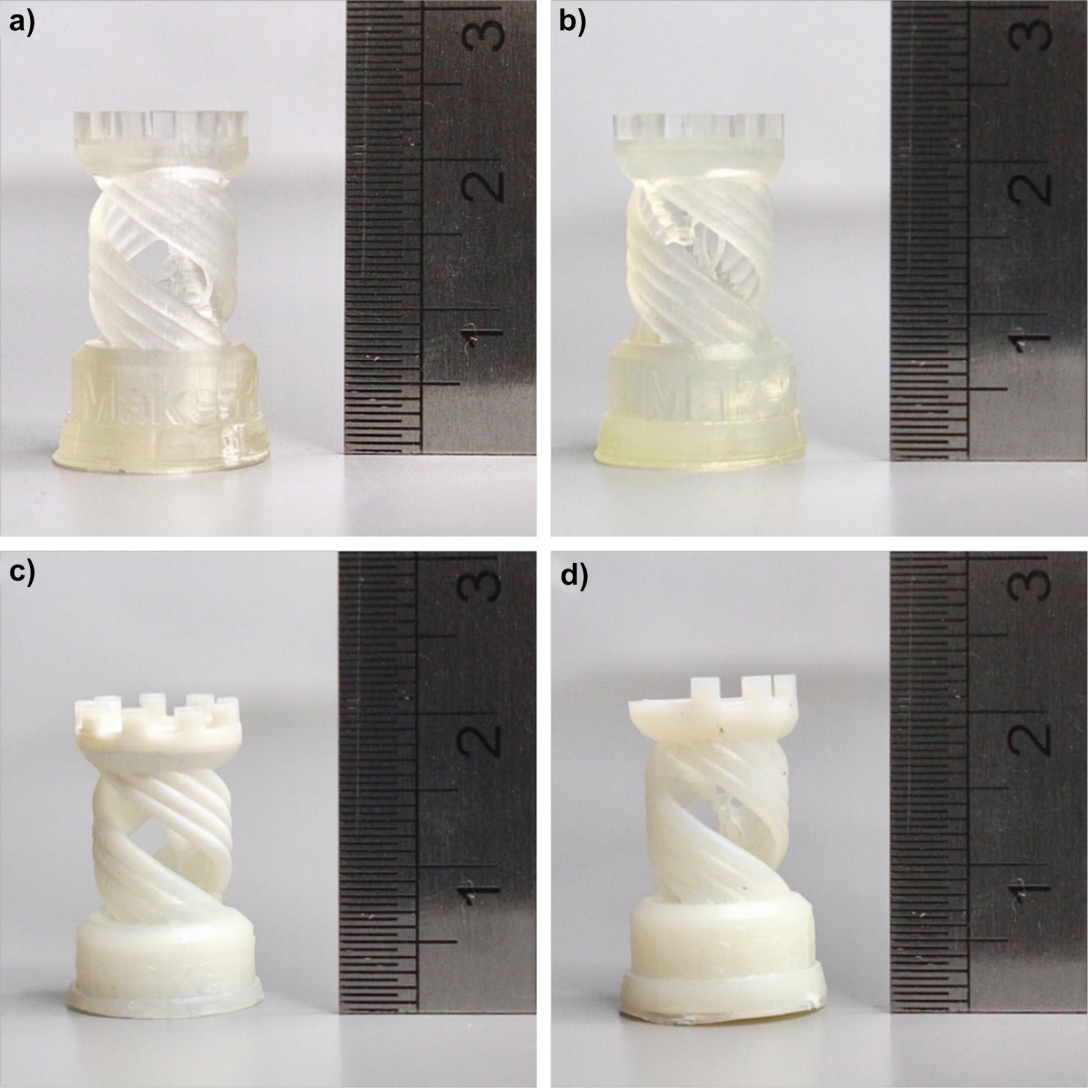
Photograph of the rock tower printed with UV-curable acrylate-based resin (**a**) and formulated UV-curable acrylate-based resins with 30%vol; (**b**) STDPPG430, (**c**) STDPPG1030, and (**d**) STDPPG2030.

**Figure 4:**
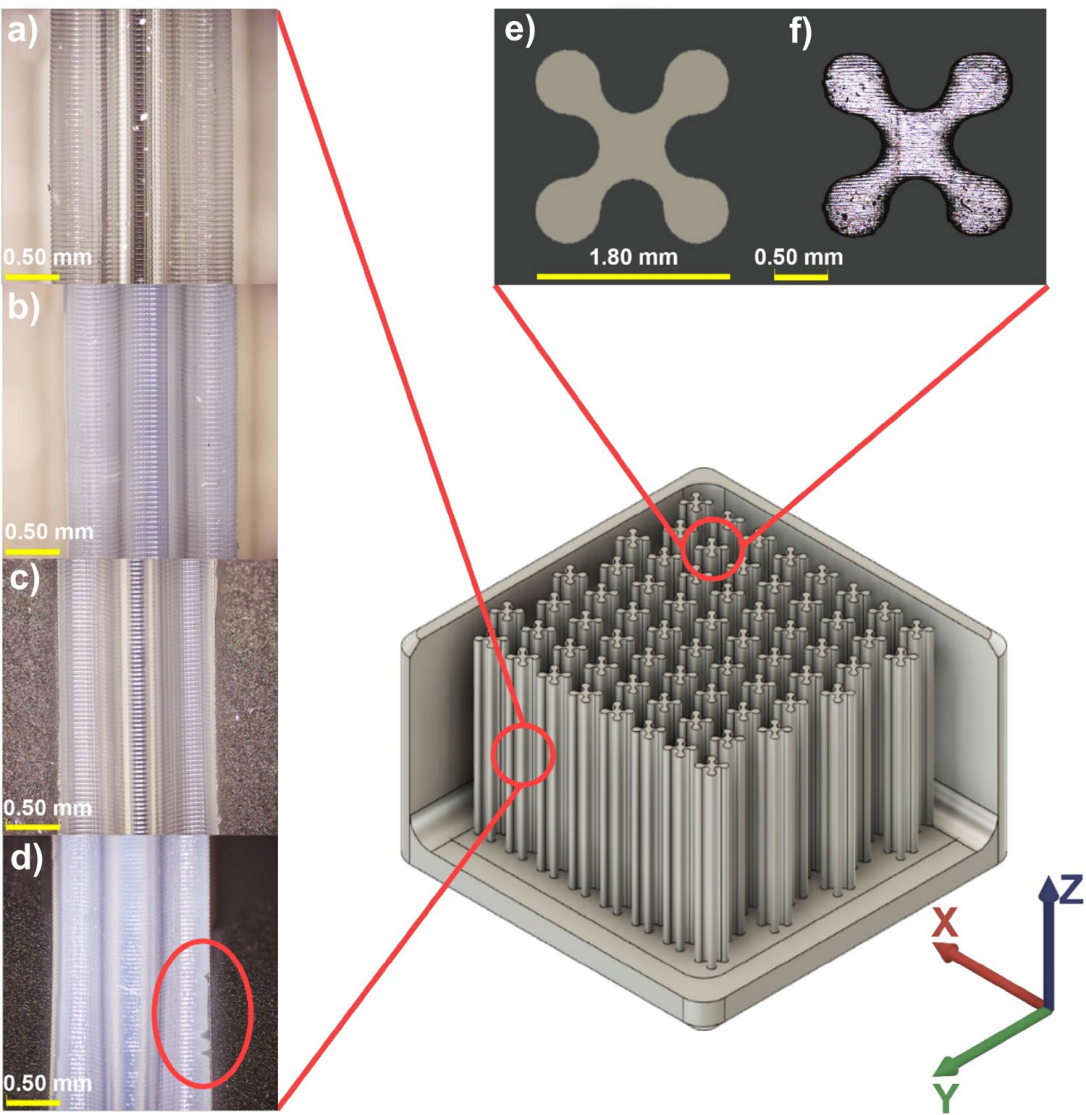
Confocal pictures of the printed stack layer (Z-axis) of the micro-reactor prototype printed from different resins; (**a**) STD, (**b**) STDPPG430, (**c**) STDPPG1030, and (**d**) STDPPG2030, and the printed layer (XY-planar); (**e**) designed pattern and (**f**) 3D printed pattern.

During printing, the acrylate-based monomers were polymerized, forming a single layer of acrylate polymer to create the desired printed model, evidencing from the decrease in intensity of C=C peak at 809 cm^−1^ could indicate the conversion of acrylate double bond to form acrylate polymer during photopolymerization of acrylate end-group^31–33^ (see Fig. S1). However, the print failure was occasionally observed in the formulated resins with more than 20%vol PPG loading, particularly the STDPPG2030. It could infer that the long-chain PPG obstructed the arrangement of the main polymer chain during photo-polymerization, causing print failure. Figure 3 shows the photograph of the rook tower prototype printed with STD, STDPPG430, STDPPG1030, and STDPPG2030, respectively. Despite the color deviation, it was observed that the dimension accuracy of STDPPG430 was nearly the same compared to the STD resin. Also, the formulated resins with PPG1000 and PPG2000 showed poor dimension accuracy, particularly the defects (the top area of the rook tower) were observed for the formulated resin with PPG2000. Moreover, the printed STDPPG1030 prototype was 8% shorter than the others since the bottom layers were peeled off during removal from the print plate. Furthermore, the micro-reactor prototypes with the tiny confinements were 3D printed with STD and formulated resins under the same printing condition to evaluate the printing accuracy of the custom-designed prototype, as shown in Fig. 4. Obvious printing defects were observed in the prototype printed from the formulated resin with PPG2000 (Fig. 4d). Moreover, the shrinkage tends to be more dominant in the X–Y direction (the width and the thickness of the printed specimen), as presented in Table S3. For the complex part shown in Fig. 4, an obvious shrinkage can be observed for the formulated resins with longer chain molecules. Normally, volumetric shrinkage can occur during polymerization due to the replacement of Van der Waals force by strong and short covalent bonds between two molecules. The multiplication of this replacement is responsible for the overall shrinkage in the material^34, 35^. The longer chain molecules, PPG1000 and PPG 2000, could obstruct the crosslinking process and lead to more volumetric shrinkage of the printed resin compared to the shorter chain molecule, PPG400.

### Dynamic mechanical analysis (DMA)

**Figure 5:**
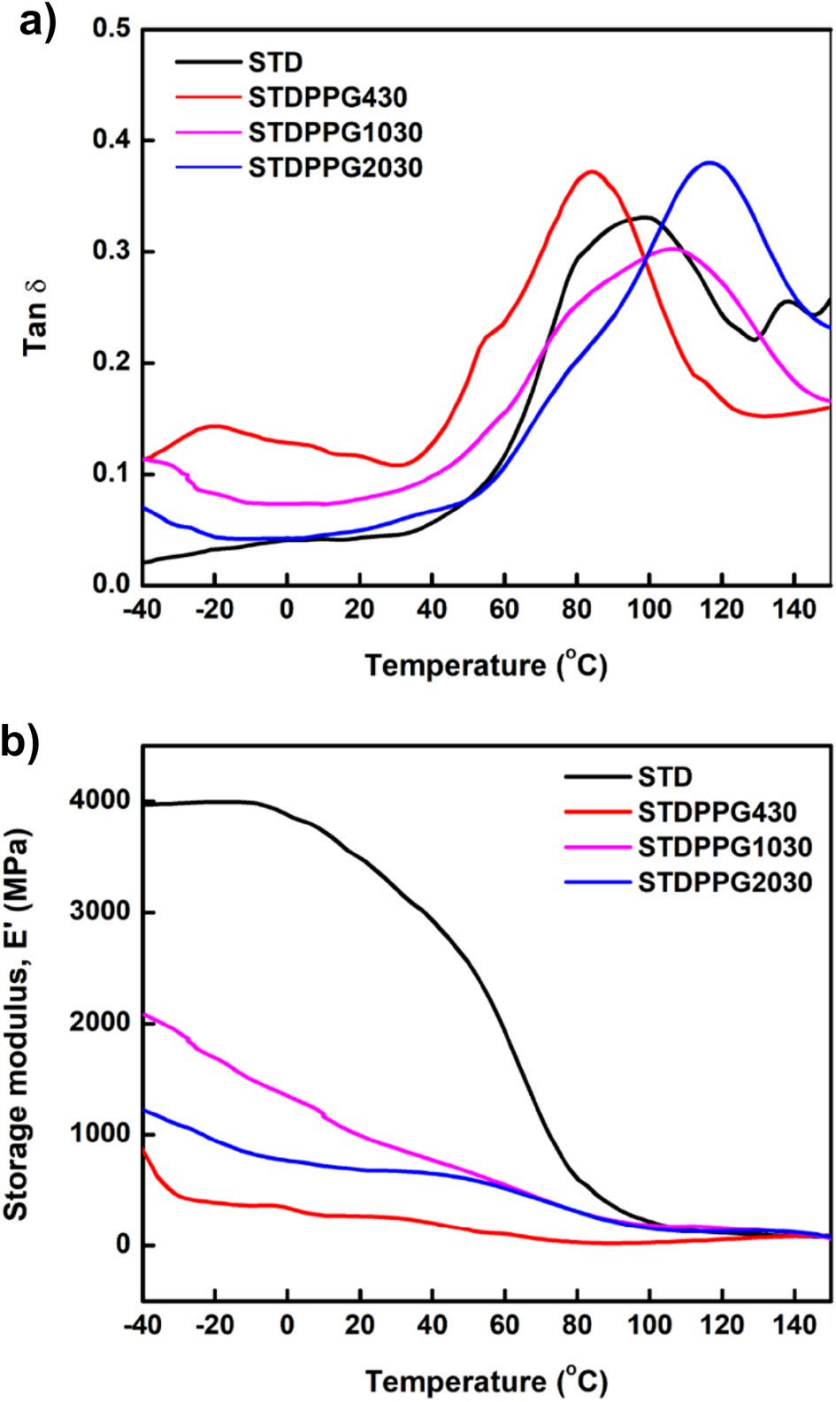
Dynamic mechanical temperature sweep curves at frequency of 5 Hz of the UV-curable acrylate-based resin (STD) and formulated resins with PPG400, PPG1000 and PPG2000 at 30%vol added; (**a**) loss factor (tanδ) and (**b**) storage modulus (E’).

To study the influence of different PPG chain lengths on the viscoelastic properties of the formulated UV-curable acrylate/PPG resins. DMA plots are depicted in Fig. 5. as the storage modulus and loss tangent (tan δ) curves with respect to temperatures. The sharp reduction in the storage modulus curve and the peak of loss tangent (tanδ) curve are indications of the structural phase transformation occurring from the relaxation of the polymer chains throughout the thermal transition. Thus, the magnitude of the tanδ can be applied to predict the characteristic glass transition temperature of materials^36–38^. Based on the peak of tanδ, the T_g_ was found to be 99.11 °C, 86.24 °C, 108.46 °C, and 113.30 °C for UV-curable acrylate-based resin and the formulated resin with PPG400, PPG1000 and PPG2000, respectively. The T_g_ of the formulated resin with PPG400 shifted gradually to the low-temperature zone compared to the pure UV-curable acrylate-based resin, indicating that the PPG400 brings more flexibility to the network of the UV-curable acrylate-based resin. A higher chain segment stiffness results in a higher T_g_^39–42^. On the other hand, the addition of the longer chain PPG1000 and PPG2000 resulted in an increase of T_g_ and decreasing of chain mobility in the polymer matrix^26^.

Storage modulus (E’) indicates the elasticity or solid-like character of materials. It is used to determine the rigidity or the ability to store energy for a certain material. The high value of E’ value refers to the greater elastic property of the material, meaning that it is more difficult to break down such material. Thus, the printed part with high storage modulus means that the part is more stable after printing^43, 44^. As shown in Fig. 5., the storage modulus (E’) of the UV-curable acrylate-based resin and the formulated resins with PPG400, PPG1000 and PPG2000 at room temperature are 3366.15 MPa, 262.51 MPa, 924.35 MPa, and 677.87 MPa respectively. It is worth noting that the PPG chain lengths influenced the viscoelastic property of the formulated resins. The shorter chain segment of PPG400 could bring about the flexibility for the formulated resin, while the longer chain segments, PPG1000 and PPG2000, led to stiffer printed products.

### Flexural properties

**Figure 6:**
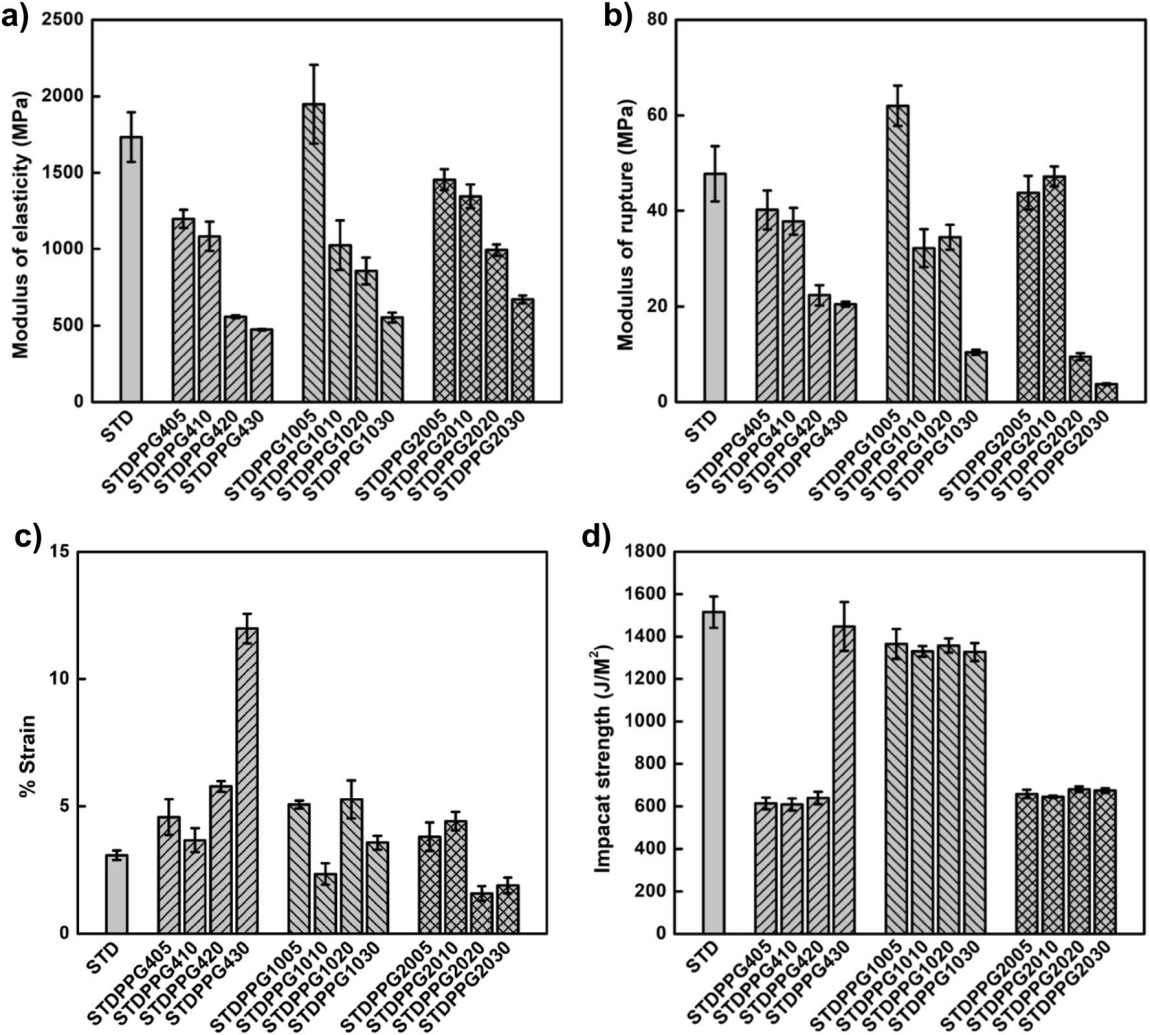
Mechanical properties of the printed STD and formulated resins; (**a**) modulus of elasticity, (**b**) modulus of rupture, (**c**) flexural strain, and (**d**) impact strength.

The flexural properties of the printed specimens from the UV-curable acrylate-based resin and the formulated resins with PPG400, PPG1000, and PPG2000 at room temperature are presented in Fig. 6. The modulus of elasticity (MOE) means the stiffness or the pressure force that a material can sustain before being permanently deformed. The results indicated that the MOE of the formulated resin with 5%vol PPG1000 improves to 1947.84 $\pm$ 258.36 MPa, compared to that of the UV-curable acrylate-based resin, 1733.90 $\pm$ 162.55 MPa. However, the MOE decreased with increasing the PPG1000 loading. Similarly, for the formulated resins with PPG400 and PPG2000, the more PPG loading, the more decrease in MOE. These phenomena were also observed in the modulus of rupture (MOR) results, which measure a material strength before rupture. The 5%vol of PPG1000 addition increased the MOR to 61.99 $\pm$ 4.21 MPa. Likewise, the increase in PPG loading led to a decrease in the MOR of the printed specimen. This may be due to the 5%vol PPG1000 acting as the nucleating agent in the UV-curable acrylate-based resin matrix, providing higher mobility to the chains, further promoting crystallization^45^. Figure 6c shows the elongational property of the printed specimens from the formulated resins. The 5–10%vol of PPG could improve the elongation of the conventional UV-curable acrylate-based resin. Remarkably, incorporation 30%vol PPG400 increased the percentage stain up to 11%. Thus, it can be said that the flexibility of the formulated resin with 30%vol PPG400 is significantly improved.

### Impact properties

The impact strength of the printed specimens is presented in Fig. 6d. The printed specimens from the formulated resin with 30%vol PPG400 exhibited the highest impact strength (1448.21 $\pm$ 115.54 J/m^2^). This again confirmed that the formulated resin with 30%vol PPG400 is significantly improved in its flexible and ductile properties.

### Hardness properties

The shore D hardness of the printed specimens is presented in Table1. The UV-curable acrylate-based resin was softened when formulated with PPG load increasing and the shorten of PPG chain segment, the formulated resin with 30%vol PPG400 exhibited the softest (hardness at 46 shore D). It can infer that PPG400 was infiltrating into the UV-curable acrylate-based resin matrix and appropriately obstructing the crosslinking, leading to the more amorphous phase in the polymer matrix, which was further reflected in a decrease of the MOE and the MOR while increasing the elongation at break and the impact strength^46, 47^.

**Table 1:** Shore hardness of formulated resin specimen.

Adding percentage	STD	PPG400	PPG1000	PPG2000
100	59D	–	–	–
5	–	55D	57D	58D
10	–	52D	54D	56D
20	–	48D	50D	52D
30	–	46D	48D	50D

## Conclusion

Regarding thermal stability and mechanical performance, the formulated resin system could be a promising candidate for fabricating flexible and reversible 3D structures in many applications, such as flexible and wearable electronic devices and soft robotics. The formulated resins with 5–30%vol PPG were successfully printed with acceptable printing accuracy at 96% (shown in Table S3). The addition of short-chain PPG400 at 30%vol led to a decrease in T_g_, thus, improving the flexibility of the printed parts. On the other hand, the addition of the longer chain PPG1000 and PPG2000 resulted in an increase of T_g_ and decreasing of chain mobility in the polymer matrix, causing the printing layer to shrink and leading to print failure and incomplete printing. With 5%vol PPG addition, the flexural strength of the specimens increased and reached 45.42 MPa. higher PPG content, leads to a decrease in the flexural strength of the sample and the same phenomena were also found for the impact strength and the hardness. It can be noted that a larger amount of PPG may have a negative effect, while the small amount of PPG successfully enhanced the mechanical performance of the printed specimens.

Also, the low-cost PPG used as filler provide a very cost-competitive material when compared to commercially available products. The successful improvement in the mechanical performance as well as the printability; the formulated resins could be applied to a variety of applications such as flexible and wearable devices, micro-fluidic devices, and small devices where high mechanical strength and complex shapes are required.

## Methods

### Resin formulation design

To improve flexibility of 3D printed specimen by adding the soft polymer in to polymer matrix. Polypropylene glycol was adding into commercial UV-curable acrylate-based resin for blending at 5%, 10%, 20%, and 30% by volume as show in Table 2. Polypropylene glycol (DL400, DL1001, and DL2001, SyncoPol^®^) was obtained from IRPC Polyol Co., Ltd. Commercial photopolymer resin was supplied by Sync Innovation Co., Ltd.

**Table 2:** Polymers blending ratio.

	UV-curable acrylate-based resin (ml)	Polypropylene glycol (ml)	Note
STD	100	0	Commercial resin (clear)
STDPPG405	95	5	DL400
STDPPG410	90	10	
STDPPG420	80	20	
STDPPG430	70	30	
STDPPG1005	95	5	DL1001
STDPPG1010	90	10	
STDPPG1020	80	20	
STDPPG1030	70	30	
STDPPG2005	95	5	DL2001
STDPPG2010	90	10	
STDPPG2020	80	20	
STDPPG2030	70	30	

### The fabrication of 3D printed specimen

Direct Light Process (DLP) 3D printer, Elegoo Mars Pro, was used to fabricate the 3D printed specimen. The test specimen was designed using Fusion 360 as CAD software based on testing standard that related to testing topic. DLP printing parameters were optimized and are listed in Table 3. It is noted that the same printing parameters were used for all polymer formulated resins resin. After completed 3D printing, all 3D printed specimens were washed with isopropyl alcohol (IPA) in an ultrasonic bath for 10 min to remove unpolymerized resin and then post-cured under a UV-lamp for 15 min.

**Table 3:** 3D printing condition.

Parameter	Value
Light source wavelength (nm)	405
Layer height (mm)	0.05
Exposure time (s)	8
Bottom exposure time (s)	45
Bottom layer count	5
Support	Enable
Anti-aliasing	Enable

### UV–VIS absorbance analysis

To measure the UV absorbance, 3D printing materials was mixed with IPA in ratios 1:100 resin to IPA prior performed using LabTech BlueStar B, UV–VIS spectrophotometer with absorbance spectra 300 to 600 nm in 1 nm steps by focusing on the absorbance on wavelength 405 nm that is the recommended condition for 3D printing.

### Thermal analysis

Thermogravimetric analysis (TGA) measurements were carried out using METTLER TOLEDO, TGA/DSC 3 + 3D printed specimens were heated from 30 °C to 600 °C, under the nitrogen atmosphere with a scanning temperature rate at 10 K/min.

### Thermomechanical analysis

Dynamic mechanical analysis (DMA) Viscoelastic properties and T_g_ of 3D printed specimens were determined using Mettler Toledo DMA/SDTA 861^e^. Samples were printed as rectangular bars (12 × 30 × 1.8 mm^3^) and placed onto the DMA with single cantilever mode at frequency 5 Hz with a heating rate 3 °C min^−1^ over a temperature range of −40 °C to 150 °C.

### Printing accuracy test

Printing accuracy can be testing by measure the dimension of CAD 3D model file compare with the dimension of 3D printed specimen then calculating as percentage. Printing accuracy was evaluated as an average value of at least three replicates.

### The flexural strength

The testing specimens were printed based on ASTM D790 geometry and performed by Tinius Olsen 5ST, universal testing machine (UTM), testing speed was set at 5 mm/min with load cell 5 kN, the flexural strength was evaluated as an average value of at least five replicates.

### The impact strength

The testing specimens were printed based on ASTM D256 geometry and performed by Gotech GT-7045-MD, impact tester with 2.75 J hammer on Izod method. The impact strength was evaluated as an average value of at least five replicates.

### The hardness

Specimens were evaluated by testing based on ASTM D2240 by using Shore Instruments & MFG. Co. durometer with shore D. The hardness was evaluated as an average value of at least five replicates.

## Supplementary Information

Supplementary Information.

## Data Availability

The datasets used and/or analyzed during the current study available from the corresponding author on reasonable request.
