# Supplementary material for: Mechanically tunable resins based on acrylate-based resin for digital light processing (DLP) 3D printing
Source: Sci Rep. 2022 Nov 21;12:20025. doi: 10.1038/s41598-022-24667-8 (PMC9681742; doi:10.1038/s41598-022-24667-8)
Supplement: Supplementary file 1 — Supplementary Information. [file 41598_2022_24667_MOESM1_ESM.docx]

**Mechanically tunable resins based on acrylate-based resin for Digital Light Processing (DLP) 3D printing.**

Aphiwat Pongwisuthiruchte^1^, Stephan T. Dubas^2^, Chuanchom Aumnate^3,4,*,+^, and Pranut Potiyaraj^1,3,4,*,+^

^1^Department of Materials Science, Faculty of Science, Chulalongkorn University, Bangkok, Thailand

^2^Petroleum and Petrochemical College, Chulalongkorn University, Bangkok, Thailand

^3^Metallurgy and Materials Science Research Institute, Chulalongkorn University, Bangkok, Thailand

^4^Center of Excellence in Responsive Wearable Materials, Chulalongkorn University, Bangkok, Thailand

*Corresponding. [Chuanchom.a@chula.ac.th](mailto:Chuanchom.a@chula.ac.th), [Pranut.p@chula.ac.th](mailto:Pranut.p@chula.ac.th)

^+^these authors contributed equally to this work

The acrylate conversion of the formulated resins was analyzed by Fourier transform infrared spectroscopy (FTIR) equipped with attenuated total reflectance (ATR) accessory (Nicolet iS50, Fourier transform infrared spectrometer). Figure S1 shows spectra of the uncured and cured formulated STDPPG405 resins. The uncured STDPPG405 resin showed characteristic peaks of C=O stretching at 1725 cm^−1^, C=C stretching at 809 cm^−1^, and C-H stretching at 1456 cm^−1^ which attributed to the presence of the acrylate group that being incorporated into the polymer network ^1,2^. After UV irradiation, the C-N stretching and bending N-H at 1538-1560 cm^−1^ and 1230-1250 cm^−1^, and C-O-C asymmetric stretching at 1120-1140 cm^−1^ confirmed the characteristics of polymerized polyurethane (PU) that is the main backbone of the STD resin. Moreover, the decrease in the intensity of C=C peak at 809 cm^−1^ could indicate the conversion of acrylate double bond to form acrylate polymer during photopolymerization of acrylate end-group^3,4,5^. However, because of the small content and low molecular weight of PPG, the characteristic peaks of PPG in the formulated resin were obscured in the presented FTIR spectra.


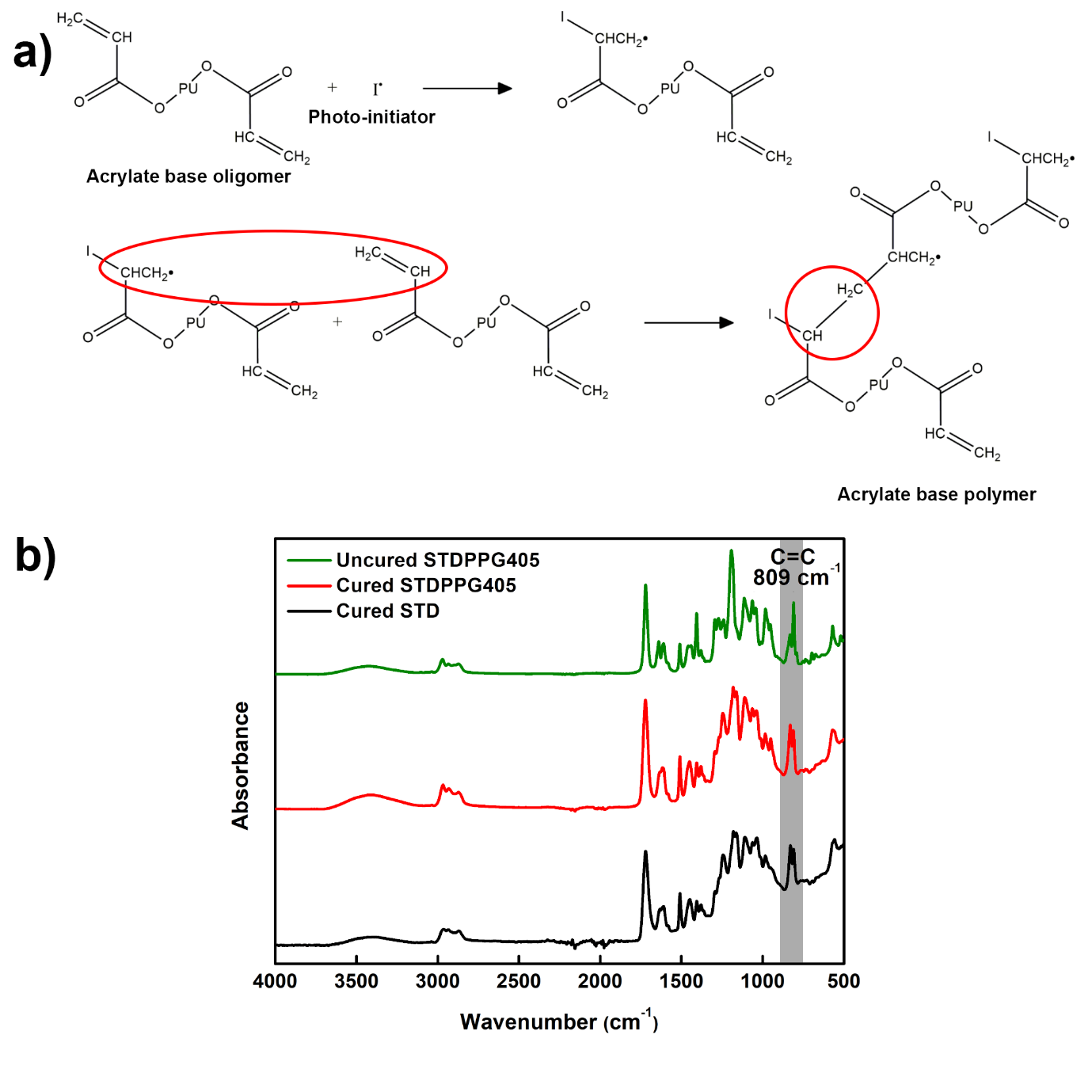


**Figure S1.** (a) Photopolymerization reaction of acrylate group and (b) FT-IR spectra of uncured STDPPG405, cured STDPPG405, and cured STD.


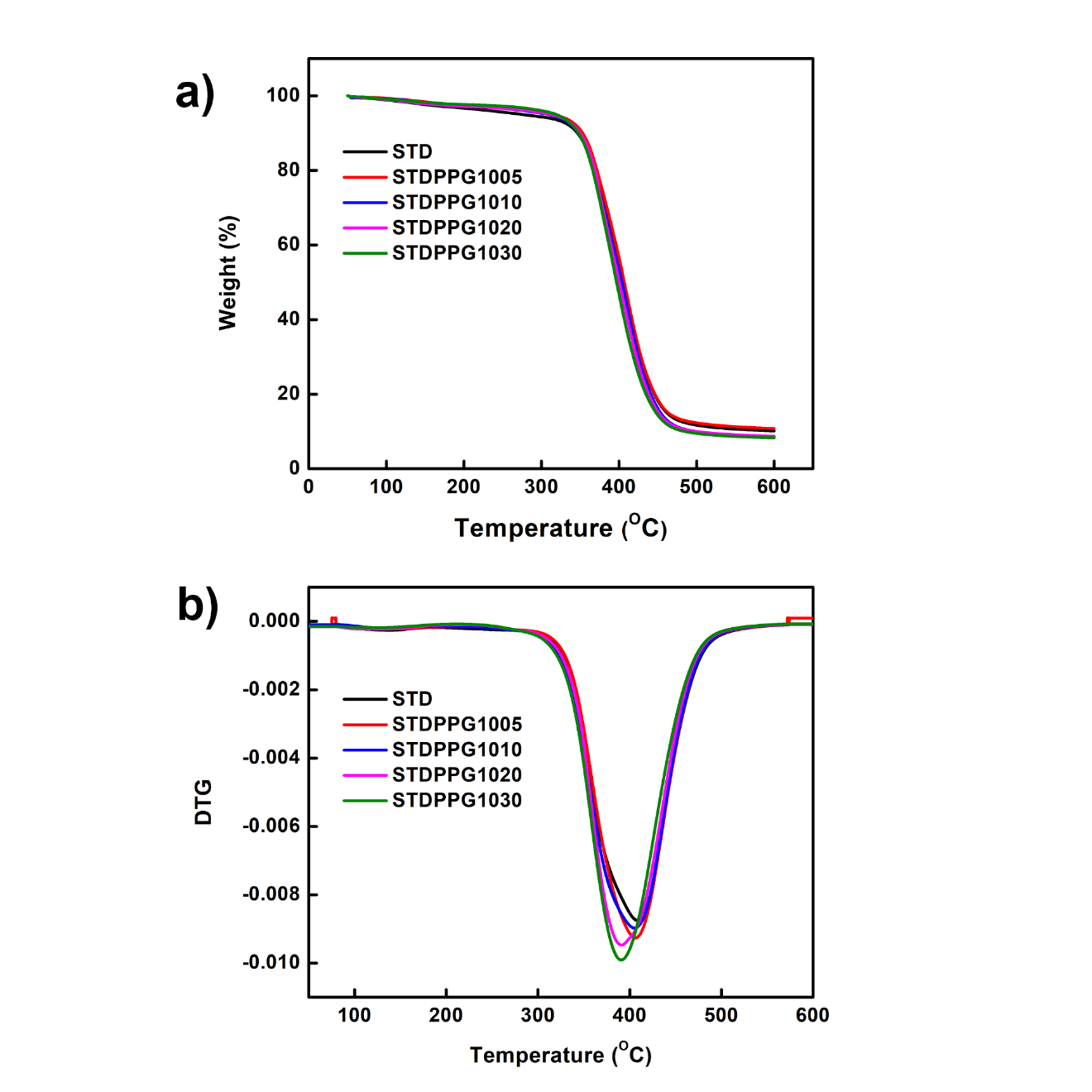


**Figure S2.** (a) Thermogravimetric analysis (TGA) curves and (b) derivative thermogravimetric analysis (DTG) curves of UV-curable acrylate-based resin (STD) and formulated resins with PPG1000.


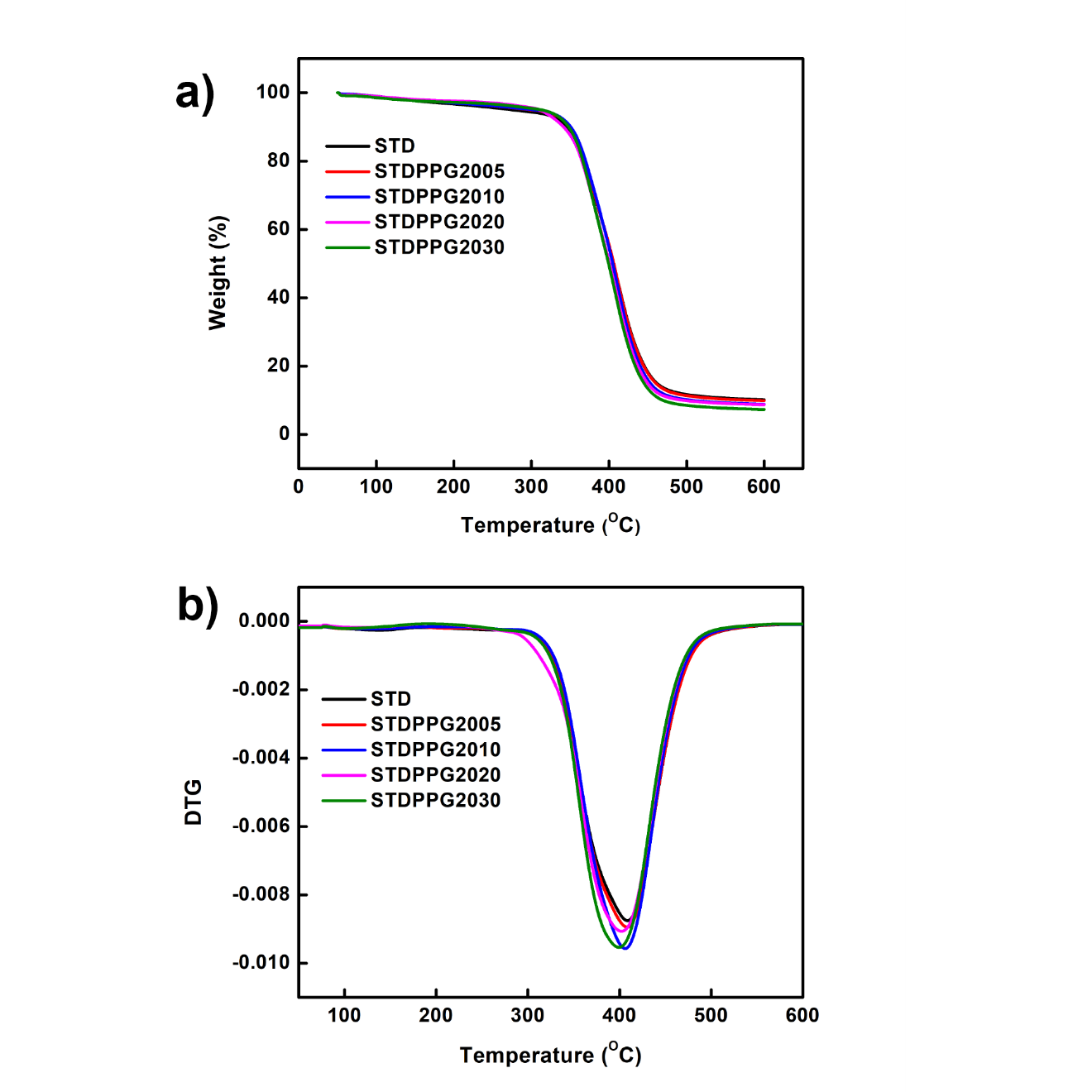


**Figure S3.** (a) Thermogravimetric analysis (TGA) curves and (b) derivative thermogravimetric analysis (DTG) curves of UV-curable acrylate-based resin (STD) and formulated resins with PPG2000.

The viscosity of PPG, UV-curable acrylate-based resin (STD), and the formulated resins with PPG were determined using a Brookfield DV-II+ viscometer at room temperature. The data is shown in Table S1. In order to prevent print failures, the resin viscosity must be kept low (250 cP for a pentaerythritol tetra-acrylate resin, 5,000 cP for ceramic suspensions, and 10,000 cP for polymer with carbon nanocomposites)^6^.

Table S1. Viscosity of PPG, UV-curable acrylate-based resin (STD) and the formulated resins with PPG

| **Materials** | **Viscosity (cP)** |
| --- | --- |
| PPG400 | 59.6 |
| PPG1000 | 130.6 |
| PPG2000 | 280.4 |
| STD | 110.4 |
| STDPPG405 | 105.5 |
| STDPPG410 | 98.4 |
| STDPPG420 | 92.7 |
| STDPPG430 | 82.7 |
| STDPPG1005 | 111.1 |
| STDPPG1010 | 115.9 |
| STDPPG1020 | 118.5 |
| STDPPG1030 | 123.4 |
| STDPPG2005 | 128.7 |
| STDPPG2010 | 129.5 |
| STDPPG2020 | 164.4 |
| STDPPG2030 | 200.0 |

The Gel content measurements were performed with a Soxhlet extractor using THF as a solvent for 24 h to completely remove the uncrosslinked constituents. The specimens removed from Soxhlet were dried in a vacuum oven for an hour. The average gel contents (wt.%) were calculated according to equation SE1; the results are shown in Table S2.

$G\text{el content }\text{ }\text{=} \frac{\text{m}\text{1}}{\text{m}\text{0}}\text{ ×}\text{ }\text{100 \%}$ (SE1)

where m_0_ is the initial sample's weight and m_1_ is the mass of extracted samples after drying in oven.

Table S2 Gel content percentage of formulated resin specimen

|  | **Gel content (%)** |
| --- | --- |
| STD | 99.54 |
| STDPPG430 | 87.40 |
| STDPPG1030 | 87.43 |
| STDPPG2030 | 87.49 |

The dimensional accuracy of 3D printed specimens was calculated according to equation SE2; the results are demonstrated in Table S2. The dimension of CAD specimen is 80 mm x 12 mm x 4 mm (length x width X thickness).

$\text{Dimension accuracy = }\frac{\text{Average dimension of printed specimen}}{\text{Dimension of CAD specimen}}\text{ ×100 \%}$ (SE2)

Table S3 Average dimensional data and dimension accuracy of 3D printed specimens from STD and formulated resins.

| **Materials** | **Average length  (mm)** | **Length accuracy  (%)** | **Average width  (mm)** | **Width accuracy  (%)** | **Average thickness (mm)** | **Thickness accuracy  (%)** |
| --- | --- | --- | --- | --- | --- | --- |
| STD | 79.76±0.02 | 99.70±0.02 | 11.82±0.02 | 98.50±0.02 | 3.94±0.02 | 98.50±0.02 |
| STDPPG405 | 79.74±0.02 | 99.68±0.02 | 11.78±0.02 | 98.17±0.02 | 3.93±0.02 | 98.25±0.02 |
| STDPPG410 | 79.70±0.02 | 99.63±0.02 | 11.77±0.02 | 98.08±0.02 | 3.92±0.02 | 98.00±0.02 |
| STDPPG420 | 79.70±0.02 | 99.63±0.02 | 11.76±0.02 | 98.00±0.02 | 3.92±0.02 | 98.00±0.02 |
| STDPPG430 | 79.70±0.02 | 99.63±0.02 | 11.76±0.02 | 98.00±0.02 | 3.92±0.02 | 98.00±0.02 |
| STDPPG1005 | 79.75±0.02 | 99.69±0.02 | 11.82±0.02 | 98.50±0.02 | 3.94±0.02 | 98.50±0.02 |
| STDPPG1010 | 79.74±0.02 | 99.68±0.02 | 11.76±0.02 | 98.00±0.02 | 3.92±0.02 | 98.00±0.02 |
| STDPPG1020 | 79.73±0.03 | 99.66±0.03 | 11.73±0.03 | 97.75±0.03 | 3.91±0.03 | 97.75±0.03 |
| STDPPG1030 | 79.70±0.03 | 99.63±0.03 | 11.63±0.04 | 96.92±0.04 | 3.88±0.03 | 97.00±0.03 |
| STDPPG2005 | 79.69±0.02 | 99.61±0.02 | 11.77±0.02 | 98.08±0.02 | 3.92±0.02 | 98.00±0.02 |
| STDPPG2010 | 79.68±0.03 | 99.60±0.03 | 11.76±0.03 | 98.00±0.03 | 3.92±0.04 | 98.00±0.04 |
| STDPPG2020 | 79.38±0.03 | 99.23±0.03 | 11.63±0.04 | 96.92±0.04 | 3.88±0.04 | 97.00±0.04 |
| STDPPG2030 | 79.35±0.04 | 99.19±0.04 | 11.54±0.04 | 96.17±0.04 | 3.85±0.04 | 96.25±0.04 |

**Reference**

1 Tzeng, J.-J., Yang, T.-S., Lee, W.-F., Chen, H. & Chang, H.-M. Mechanical Properties and Biocompatibility of Urethane Acrylate-Based 3D-Printed Denture Base Resin. *Polymers* **13**, 822 (2021).

2 Qin, L. *et al.* Preparation and properties of polyurethane acrylates modified by saturated alcohols. *Progress in Organic Coatings* **76**, 1594-1599 (2013). <https://doi.org:10.1016/j.porgcoat.2013.07.005>

3 Sonpatki, M. M., Sergan, T., Kelly, J. & Chien, L. C. Anisotropic photo-polymerization and fluorescence study of discotic materials. *Macromolecular Symposia* **154**, 83-94 (2000). <https://doi.org:https://doi.org/10.1002/1521-3900(200004)154:1><83::AID-MASY83>3.0.CO;2-X

4 Liu, J. *et al.* Synthesis of UV-curable polycarbonate diols (PCDL)-based polyurethane acrylate for negative photoresist. *Polymer Bulletin* **73**, 647-659 (2015). <https://doi.org:10.1007/s00289-015-1511-4>

5 Zhang, D. *et al.* Preparation and properties of UV-curable waterborne silicon-containing polyurethane acrylate emulsion. *Progress in Organic Coatings* **160** (2021). <https://doi.org:10.1016/j.porgcoat.2021.106503>

6 Luo, Y., Le Fer, G., Dean, D. & Becker, M. L. 3D Printing of Poly(propylene fumarate) Oligomers: Evaluation of Resin Viscosity, Printing Characteristics and Mechanical Properties. *Biomacromolecules* **20**, 1699-1708 (2019). <https://doi.org:10.1021/acs.biomac.9b00076>
